# Unravelling the Potential Cytotoxic Effects of Metal Oxide Nanoparticles and Metal(Loid) Mixtures on A549 Human Cell Line

**DOI:** 10.3390/nano10030447

**Published:** 2020-03-02

**Authors:** Fernanda Rosário, Maria João Bessa, Fátima Brandão, Carla Costa, Cláudia B. Lopes, Ana C. Estrada, Daniela S. Tavares, João Paulo Teixeira, Ana Teresa Reis

**Affiliations:** 1 EPIUnit—Instituto de Saúde Pública, Universidade do Porto, Rua das Taipas, n° 135, 4050-600 Porto, Portugal; fernanda.rosario@insa.min-saude.pt (F.R.); m.joao.bessa@insa.min-saude.pt (M.J.B.); m.fatima.brandao@insa.min-saude.pt (F.B.); carla.trindade@insa.min-saude.pt (C.C.); joao.teixeira@insa.min-saude.pt (J.P.T.); 2 Department of Environmental Health, Portuguese National Institute of Health, Rua Alexandre Herculano, 321, 4000-055 Porto, Portugal; 3 ICBAS—Institute of Biomedical Sciences Abel Salazar, U. Porto—University of Porto, Rua Jorge de Viterbo Ferreira 228, 4050-313 Porto, Portugal; 4 Department of Chemistry and Aveiro Institute of Materials (CICECO), University of Aveiro, Campus de Santiago, 3810-193 Aveiro, Portugal; claudia.b.lopes@ua.pt (C.B.L.); ana.estrada@ua.pt (A.C.E.); danielatavares@ua.pt (D.S.T.); 5 Department of Chemistry and Center of Environmental and Marine Studies (CESAM), University of Aveiro, Campus de Santiago, 3810-193 Aveiro, Portugal; 6 CIIMAR, Interdisciplinary Centre of Marine and Environmental Research, University of Porto, Terminal de Cruzeiros do Porto de Leixões, Avenida General Norton de Matos, S/N, 4450-208 Matosinhos, Portugal

**Keywords:** cytotoxicity, co-exposure, mixtures, A549, arsenic, mercury, titanium dioxide nanoparticles, cerium oxide nanoparticles, clonogenic assay, cell-cycle

## Abstract

Humans are typically exposed to environmental contaminants’ mixtures that result in different toxicity than exposure to the individual counterparts. Yet, the toxicology of chemical mixtures has been overlooked. This work aims at assessing and comparing viability and cell cycle of A549 cells after exposure to single and binary mixtures of: titanium dioxide nanoparticles (TiO_2_NP) 0.75–75 mg/L; cerium oxide nanoparticles (CeO_2_NP) 0.75–10 μg/L; arsenic (As) 0.75–2.5 mg/L; and mercury (Hg) 5–100 mg/L. Viability was assessed through water-soluble tetrazolium (WST-1) and thiazolyl blue tetrazolium bromide (MTT) (24 h exposure) and clonogenic (seven-day exposure) assays. Cell cycle alterations were explored by flow cytometry. Viability was affected in a dose- and time-dependent manner. Prolonged exposure caused inhibition of cell proliferation even at low concentrations. Cell-cycle progression was affected by TiO_2_NP 75 mg/L, and As 0.75 and 2.5 μg/L, increasing the cell proportion at G0/G1 phase. Combined exposure of TiO_2_NP or CeO_2_NP mitigated As adverse effects, increasing the cell surviving factor, but cell cycle alterations were still observed. Only CeO_2_NP co-exposure reduced Hg toxicity, translated in a decrease of cells in Sub-G1. Toxicity was diminished for both NPs co-exposure compared to its toxicity alone, but a marked toxicity for the highest concentrations was observed for longer exposures. These findings prove that joint toxicity of contaminants must not be disregarded.

## 1. Introduction

Nanotechnology is an innovative scientific and economic growth area with exponential production, due to its multidisciplinary applications and economic importance. Nanoparticles (NPs) can be found in everyday products and, consequently, humans are inevitably and constantly exposed to them [1,2]. Despite their versatile application and benefits, the effect and impact of NPs on the environment and public health is still uncertain [3].

So far, nanotoxicity assessments usually focus on single NPs effects, overlooking interactions with other contaminants. However, in the environment, NPs co-exists as complex mixtures, with different types of contaminants and toxic species, such as metal(loids) [4]. Due to their surface reactivity compared to their bulk counterparts, NPs can interact with toxic compounds, and it can both amplify the toxic effect [5,6,7] and/or have a positive role in the environment [8,9,10]. For example, two of the possible scenarios are: (1) NPs may adsorb the contaminant decreasing its availability, resulting in a reduction of contaminant uptake by organisms; or (2) NPs can act as “Trojan Horses” facilitating the transport into cells or organisms, increasing the cellular dose and amplifying the toxic effect of the contaminant. In this case, the deleterious effect could be caused by either NPs or the other contaminants, or even in a synergistic way [11,12].

Titanium dioxide nanoparticles (TiO_2_NP) are among the most abundantly used NPs, inevitably raising concern about their safety profile. To date, there is a conflict about the toxicological impacts of these nanoparticles. While numerous experimental and epidemiological data confirm the relatively low toxicity of TiO_2_NP [13,14], other studies demonstrated the cytotoxic, genotoxic and carcinogenic potential of in vitro and in vivo systems [15,16,17,18]. More specifically, they have been shown to induce allergen-independent histamine release, oxidative stress on brain cells, increased micronucleus formation, DNA breakage in blood lymphocytes and lymphoblast cells, and the generation of reactive oxygen species (ROS) [19,20,21,22,23].

Cerium oxide nanoparticles (CeO_2_NP) are a rare metal oxide catalyst which has been receiving more attention in nanomedicine, increasing the likelihood of human exposure [24]. While being highly biocompatible, as well as exerting a cytoprotective effect against radiation damage, oxidative stress, and inflammation [25,26], the exposure to CeO_2_NP has otherwise been proven to have an adverse effects on lungs, spleen, liver and kidneys [27,28,29].

Among environmental contaminants, mercury (Hg) and arsenic (As) constitute a serious threat, due to their non-biodegradable, persistent, and extremely toxic character. Mercury is a potent neurotoxin that also affects the liver, kidneys and lungs, and has high teratogenic potential [30,31]. Arsenic exposure has been associated with an increased risk of cardiovascular abnormalities, neurotoxicity, nephrotoxicity, and hepatotoxicity [32]. Moreover, studies demonstrated carcinogenicity (International Agency for Research on Cancer, IARC Group 1) and genotoxicity due to oxidative stress, chromosomal aberrations, altered expression of growth factors, DNA repairing mechanisms and methylation patterns [32,33]. Although As and Hg are potentially toxic to humans its actual toxicity varies with speciation, with inorganic species being more toxic than organic ones for the first, and the opposite for the latter.

The fact that NPs have been used in metal(loid) removal from aqueous and organic solutions [22] implies a NP-metal(loid) interaction, which can also occur in biological systems. Although very limited data is found in the literature, the available evidence suggests that contaminants co-exposure may induce significant changes in their toxicity. CeO_2_NP and TiO_2_NP were found to increase lead [34] and arsenic [35] toxicity on *Ceriodaphnia dubia*. Wang et al. [36] observed a synergistic effect with binary mixtures of TiO_2_NP and As after 24 h exposure, where non-toxic concentrations (up to 50 µg/mL) of TiO_2_NPs increased the genotoxicity of As (III) (2 µg/mL) in human-hamster hybrid cells. However, some studies reported the opposite behavior. For example, Tsai et al. [37] exposed Human bronchial epithelium cells (BEAS-2B) for 24 h, to CeO_2_NPs and TiO_2_NPs in concentrations up to 500 μg/mL. In this study, CeO_2_NP were able to reduce cytosolic Ca^2+^ and mitochondrial damage caused by TiO_2_NP. In addition, Zeng et al. [38] demonstrated that CeO_2_NP (250 mg/L) had a high capacity for As (III) (0.5 ng/mL) adsorption which resulted in reduced toxicity to human bronchial epithelial cells. These conflicting results may be attributed to the time of exposure, concentration, specific interactions between NPs and metals and physicochemical parameters of the surrounding medium. Furthermore, most of the mixtures’ toxicity experiments carried out so far have examined a narrow concentration range, which is higher than the environmental expected and/or relevant, as well as under a relatively short time of exposure, on the contrary of what is observed in real scenarios.

Therefore, the aim of this work was to evaluate and compare the toxicological effects of TiO_2_NP, CeO_2_NP, As, and Hg, as single exposures or as binary mixtures, on A549 lung cells. For that purpose, the present study aims to: (I) evaluate A549 cell viability and proliferation after short (24 h) and long-term (seven days) exposures; and (II) analyze possible interferences in cell-cycle dynamics.

## 2. Materials and Methods

### 2.1. Nanoparticles and Chemicals

Cerium oxide NPs (CAS No. 1306-38-3; #643009; <25 nm,) and Titanium dioxide NPs (CAS No. 13463-67-7; #700347; <150 nm—anatase/rutile ca. 80:20, were purchased from Sigma-Aldrich (St. Louis, MO, USA). TiO_2_NP and CeO_2_NP were first suspended in distilled water (ddH_2_O), vortexed for 1 min, dispersed by ultrasonic vibrations in an ultrasonic bath (Bandelin Sonorex RK100) (St. Louis, MO, USA) for 15 min, and vortexed for another minute. This procedure proved to result in a stable suspension (data not shown). Nanoparticles were then diluted in complete culture medium to the desired concentrations according to the test to be performed. Sodium meta-arsenite (NaAsO_2_, ≥90%, CAS No. 7784-465-56) and mercury(II) chloride (HgCl_2_; CAS No. 7487-94-7) were purchased from Sigma-Aldrich (Mannheim, Germany) and Merck (St. Loius, MO, USA), respectively. Sodium meta-arsenite is among the most common arsenic trivalent compounds, is the active ingredient in many herbicides, defoliants, insecticides, preservatives, antiseptics, soaps, among other products, and therefore humans are exposed to it through inhalation or skin absorption. Mercury chloride is one of the most toxic forms of mercury due to its high solubility in water. Solutions of both chemicals were prepared in complete culture medium to the desired concentrations and vortexed before use. Thiazolyl Blue Tetrazolium Bromide (MTT; CAS No. 298-93-1), Triton X-100 (CAS No. 9002-93-1), and Propidium Iodide (PI; CAS No. 25535-16-4) were purchased from Sigma-Aldrich (Sintra, Portugal). Cell Proliferation Reagent water-soluble tetrazolium (WST-1) and Trypsin-ethylenediaminetetraacetic acid (EDTA) 0.25% were purchased from Merck. Dimethyl sulfoxide (DMSO; CAS No. 67-68-5) and RNase (DNase free; CAS No. 9001-99-4) were obtained from Honeywell (Seelze, Germany) and PanReac AppliChem (Castellar del Vallès, Barcelona), respectively. Dulbecco minimal Eagle’s medium (DMEM) was from Lonza (Alfragide, Portugal); Fetal bovine serum (FBS) and Antibiotic-Antimycotic (100×) were from Gibco (Porto, Portugal).

### 2.2. Nanoparticles Characterization

X-ray powder diffraction (XRD) data were collected using a PANanalytical Empyrean X-ray diffractometer (PANanalytical, Almelo, The Netherlands) equipped with Cu-Kα monochromatic radiation source (λ = 1.54178 Å) at 45 kV/40 mA. The samples were prepared by the deposition of aliquots of the ethanolic suspensions of TiO_2_NP or CeO_2_NP on a silicon holder. For transmission electron microscopy (TEM) analysis, a drop of the aqueous suspension under analysis was placed on a carbon-coated Cu grid and the solvent was left to evaporate at room temperature. TEM was performed using a scanning transmission electron microscope Hitachi—HD 2700, operating at 200 kV. The high-resolution TEM images were acquired by using a JEOL 2200FS HR-TEM instrument (JEOL, Tokyo, Japan). Energy-dispersive X-ray spectroscopy (EDX) microanalysis was performed using an Oxford model INCA Energy TEM 250 (High Wycombe, UK). The hydrodynamic diameter and polydispersity index values (PdI) of NPs in water and complete culture medium were measured by Dynamic light scattering (DLS) using a Malvern Zetasizer Nano ZS (Malvern Instruments, Worcestershire, UK) 0 h (immediately after preparation), 6 and 24 h after preparation of the solutions (incubated at 37 °C; 5% CO_2_). Additionally, using the same equipment, surface charge (zeta-potential) of NPs in water solution was measured by laser diffractometry. Absorption maxima of NPs solutions in water and medium were scanned at 200–800 nm by UV–Vis spectrophotometer (UNICAM, Lisboa, Portugal) to check for agglomeration. All measurements were run in triplicate.

### 2.3. Cell Culture

Lung epithelial A549 cell line (ECACC 86012804; Human Caucasian lung carcinoma) was purchased from the European Collection of Authenticated Cell Cultures (ECACC, St. Louis, MO, USA ). A549 cell line was cultured in complete growth medium (DMEM with 2 mM L-Glutamine, supplemented with 10% (v/v) fetal bovine serum (FBS), antibiotic-antimycotic solution (100 units/mL of penicillin, 100 µg/mL of streptomycin). Cells were grown at 37 °C, 5% CO_2_ in a humidified atmosphere. When confluence was reached (circa 80%), cells were detached using 0.25% trypsin-EDTA and, depending on the assay, cells were seeded in 96, 24, or 6 well plates and left 24 h for adhesion. After this period, the culture medium was replaced with fresh complete medium containing single or binary exposure solutions and the respective negative and positive controls for each assay.

### 2.4. Cell Viability

MTT and WST-1 assays were performed to assess the effect on mitochondrial dehydrogenase activity after 24 h exposure to single and binary mixtures. Cells were seeded onto 96-well culture plates (1.5 × 10^5^ cells per well) and exposed to TiO_2_NP (0.75–75 mg/L), CeO_2_NP (0.1–10 μg/L), As (0.75–2.5 mg/L), and Hg (5–100 mg/L). The concentration range spanned from non-cytotoxic levels to levels where effects were found. For binary mixtures two concentrations were chosen based on the results of single exposure: TiO_2_NP (1 and 75 mg/L), CeO_2_NP (0.1 and 10 μg/L), As (0.75 and 2.5 mg/L) and Hg (10 and 20 mg/L). These correspond to (1) a concentration were no toxic effects were seen; and (2) a concentration where the substance-induced observable toxic effects. Mixture solutions were prepared in complete culture media and left in an orbital shaker for 24 h, at room temperature, to allow for adsorption equilibrium of metals to NPs [39,40]. Triton X-100 1% (24 h exposure) was used as the positive control in both assays.

At the end of the exposure, for MTT assay, cells were treated with 100 μL/well of 1 mg/mL solution of MTT and incubated for 4 h at normal culture conditions, to allow the formation of MTT formazan crystals. The medium was then replaced for 200 μL of DMSO and the plates were placed on a shaking plate for 15 min, in the dark, to solubilize the formazan crystals, as previously done by [41,42]. For the WST-1 assay similar experimental sets were maintained. After incubation cells were treated with 10 µL of WST-1 and incubated for 2 h at normal culture conditions. Optical density (OD) was read on SpectraMax^®^ iD3 multi-mode microplate reader (Molecular Devices) at 570 nm, and 450 nm for MTT and WST (reference wavelength 650 and 630 nm) respectively. The OD values obtained from the sample blanks were deducted from the OD values obtained after the assays. The values obtained thereafter have been expressed as a percentage compared to control, ((ODsample − ODblank)/(ODcontrol − ODblank) × 100). All experiments were performed at least in triplicate on three separate occasions. Data are presented as mean ± standard deviation.

The interference of NPs and metals in the assays was eliminated by reading sample blanks (cell-free) for all the concentrations tested. In preliminary experiments, the interference of FBS was tested and proved not to yield different results from serum-free medium (data not shown).

### 2.5. Clonogenic Assay

A colony formation assay (i.e., clonogenic assay) was performed as described previously [43]. Briefly, A549 cells were seeded at 100 cells/well in 6-well plates and after 24 h for cell adhesion they were exposed for seven days to single or binary mixtures of CeO_2_NP, TiO_2_NP, As and Hg (same concentrations as before). After exposure, cells were washed with PBS, fixed and stained with 0.5% crystal violet in ethanol (Merck). Colonies with more than 100 cells were counted. Plating efficiency (PE) and surviving factor (SF) were calculated. PE is the average of three independent scores for exposure vs. control, divided by the number of cells plated; SF is expressed as a percentage and it is determined by dividing the PE of the exposed cells by the PE of the controls, and then multiplying by 100.

### 2.6. Cell Cycle Analysis

Cell cycle analysis was performed according to Oliveira et al. [44], with minor modifications. Cells were seeded in 24-well plates, and after 24 h for adhesion were exposed for additional 24 h to two concentrations of NPs and metals, and also to binary mixtures of NPs and metals (same concentrations as before). For each concentration, three replicates were used. At the end of the exposure period, cells were trypsinized and centrifuged at 300 g for 5 min. The supernatant was discarded, and the cultures were washed with PBS pH 7.2. Cells were then resuspended in cold 70% ethanol for fixation and stored at −20 °C until further analysis. At the time of analysis, cells were centrifuged at 300 g for 5 min and resuspended in 1 mL PBS. Then, 50 μg/mL of propidium iodide (PI) and 50 μg/mL of RNAse were added to assure that only nuclear DNA was stained. Samples were incubated for 15 min in the dark. Relative fluorescence intensity was measured in a Guava easyCyte 8HT Benchtop Flow Cytometer (Luminex). Acquisitions were made using Guava Podemos tirar daquiSoftware (Incyte, Merck KGaA, Darmstadt, Germany). For each sample, the number of events reached approximately 5000. Debris and doublets were excluded by the definition of the specific region (area vs. red-laser peak height). Cell cycle analysis was then conducted based on the histogram outputs. The percentage of nuclei in each phase of the cell cycle (*G*0/*G*1, *S* and *G*2 phases) was analyzed using Guava InCyte Software.

### 2.7. Statistical Analysis

For all experiments, at least three replicates and three independent assays were performed and results are expressed as a mean ± standard deviation (SD). Statistical significance of the data against negative control was analyzed by one-way ANOVA followed by Dunn’s or Dunnett post-hoc test for nonparametric or parametric data, respectively, using the SigmaPlot version 12 software (Systat Software Inc., San Jose, CA, USA). Statistical differences between single and binary exposures were assessed by one-way ANOVA with Holm–Sidak post-hoc method. The differences were considered statistically significant at *p* < 0.05. Graphs were computed using software Prism 6 (GraphPad Software, San Diego, CA)

## 3. Results

### 3.1. Characterization of TiO_2_ and CeO_2_ Nanoparticles

The TiO_2_NP and CeO_2_NP were characterized for their size, shape, hydrodynamic diameter and zeta potential in their native state (purchased as a dispersion in H_2_O), prior to cell culture experimentation.

**Figure 1:**
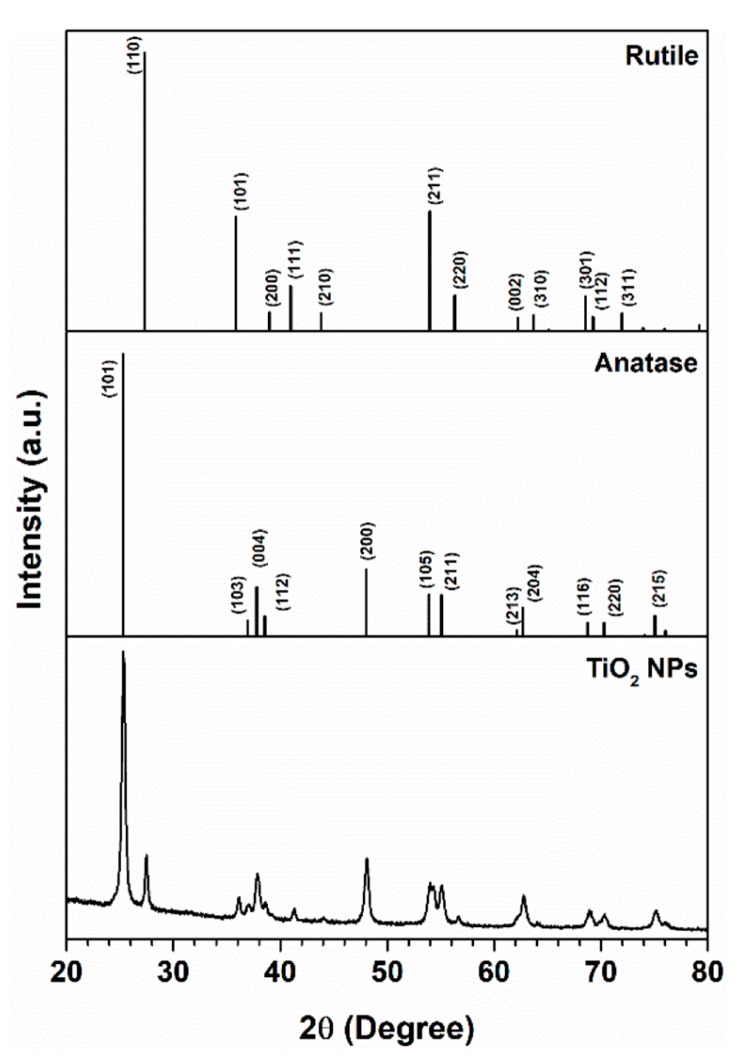
X-ray powder diffraction (XRD) patterns for TiO_2_NP. The vertical bars correspond to the diffraction peaks attributed to TiO_2_ tetragonal phases: anatase (ICDDPDF file no. 03-065-5714) and rutile (ICDDPDF file no. 04-008-7850).

The crystal structure of TiO_2_NP was studied, and the results are shown in Figure 1. The XRD patterns for the TiO_2_NP used show that they consist of biphasic TiO_2_NP containing around 80/20 wt.% of anatase/rutile. All diffraction peaks for the TiO_2_NP were ascribed to the Bragg reflections of the tetragonal phase, which are in good agreement with the TiO_2_-anatase phase (International Centre for Diffraction Data Powder Diffraction File (ICDDPDF) No. 03-065-5714) and TiO_2_-rutile phase (ICDDPDF N° 04-008-7850). There is no evidence of amorphous materials and impurities in the sample. In fact, in accordance with the XRD analysis, the energy-dispersive X-ray spectroscopy (EDX) measurements showed only the peaks for Ti of the respective TiO_2_NP. The Cu peak observed in the spectrum is attributed to the Cu grid (Figure S1).

**Figure 2:**
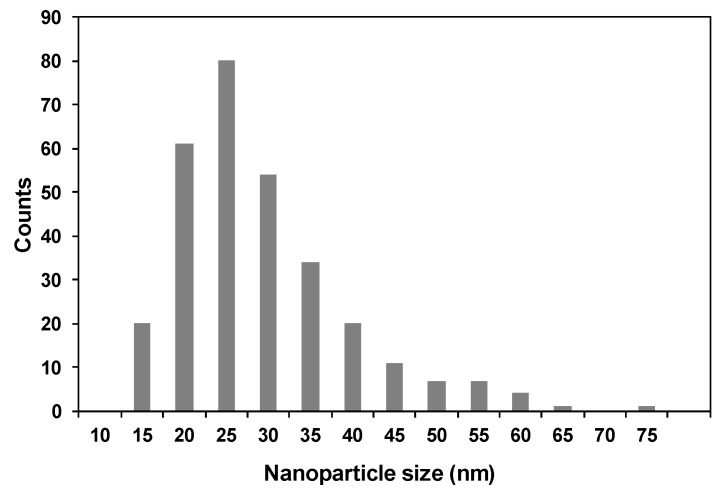
Particle size distribution histogram of TiO_2_NP.

**Figure 3:**
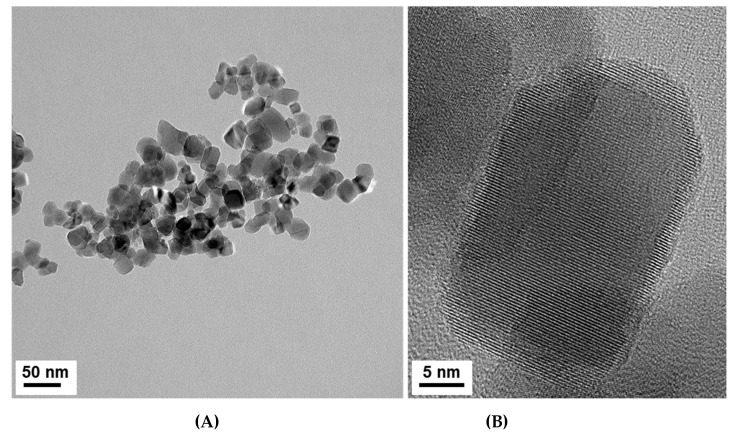
HRTEM images of TiO_2_NP (**A**) and a zoomed in detail (**B**).

The TEM analysis shows TiO_2_NP with a heterogeneous morphology and an average size particle of 26.6 ± 10.3 nm with a unimodal size distribution, which is in accordance with the average crystallite size calculated by applying the Debye–Scherrer (Figure 2). The inspection of the high-resolution TEM images confirms the presence of crystalline particles of TiO_2_, as illustrated in Figure 3, by the presence of lattice fringes of TiO_2_ and corresponding selected area electron diffraction (SAED) patterns of TiO_2_NP (Figure S2).

**Figure 4:**
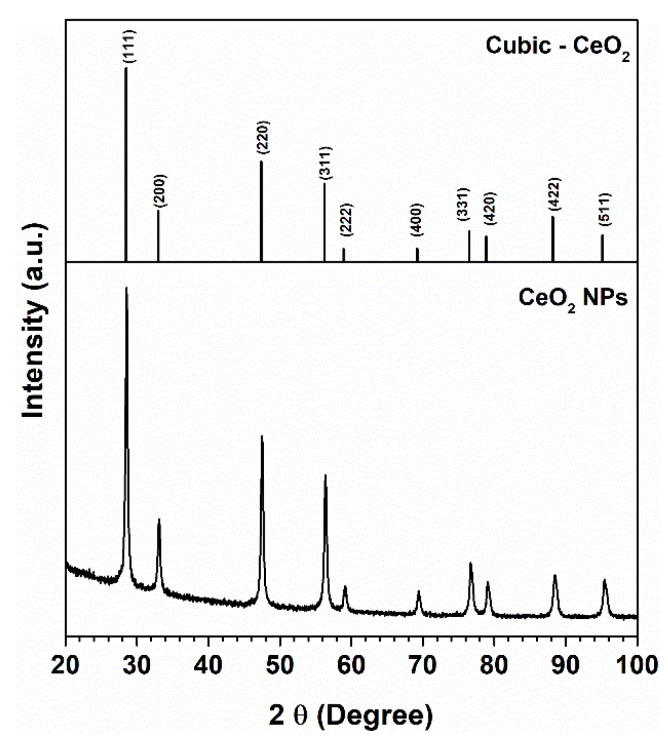
XRD patterns for CeO_2_NP. The vertical bars correspond to the diffraction peaks attributed to CeO_2_ cubic phase (ICDDPDF file no. 00-067-0123).

The XRD pattern for CeO_2_NP is shown in Figure 4. The 2θ peaks observed match with the (111), (200), (220), (311), (222), (400), (331), (420), (422) and (511) crystalline planes of the cubic structure of CeO_2_ and are in good agreement with the ICDDPDF file no. 00-067-0123. As in TiO_2_NP, the XRD pattern is suggestive of a polycrystalline material, not having evidence of amorphous impurities. Indeed, the EDX measurements performed on CeO_2_NP have shown only the presence of Ce and O from CeO_2_NP and peaks for C and Cu attributed to the copper grid, confirming the purity of samples (Figure S3).

**Figure 5:**
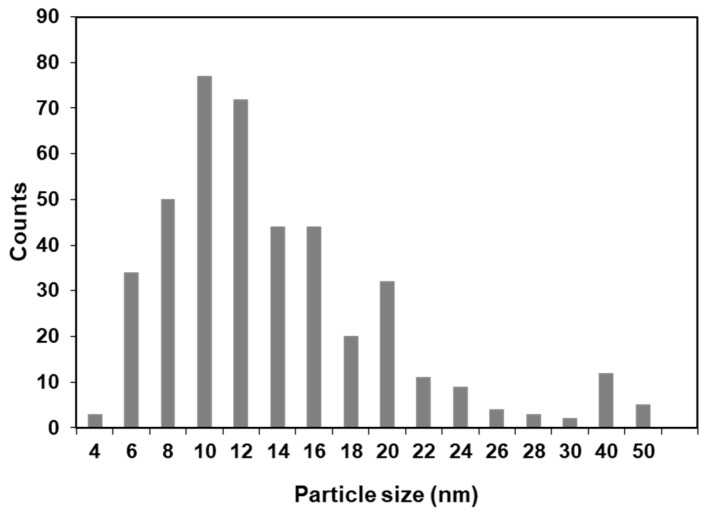
Particle size distribution histogram of CeO_2_NPs obtained from transmission electron microscopy (TEM) images.

**Figure 6:**
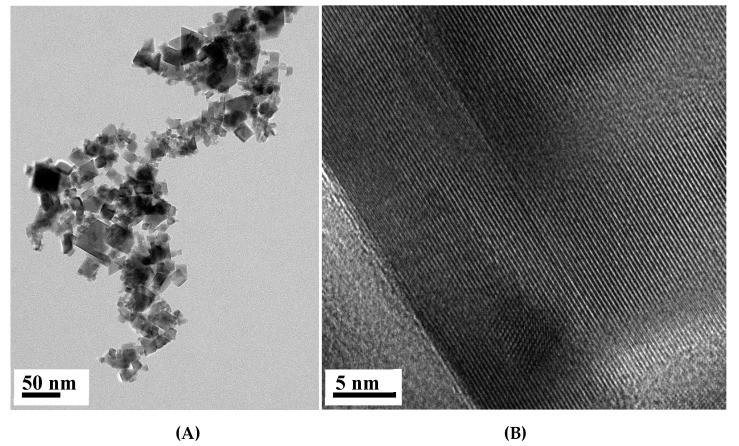
HRTEM image of CeO_2_NP (**A**) and a zoomed in detail (**B**).

The average particle size of the CeO_2_NP calculated from the half-width of the diffraction peaks and according to the Scherrer equation was 14 nm, which was also confirmed by the particle size distribution obtained by the TEM images (Figure 5). CeO_2_NPs exhibit cubic morphology, and the samples analyzed confirmed the crystalline nature of the CeO_2_ phases, which are particularly evident by inspection of the high-resolution transmission electron microscopy (HRTEM) images in Figure 6. The corresponding SAED pattern was consistent with the TEM images confirming the good crystallinity of CeO_2_NP (Figure S4).

The nanomaterials were characterized for their hydrodynamic size and polydispersity index (PdI) by DLS, after all, steps of NPs’ solutions preparation for cell exposure. Dispersion in water and immediately after sonication yielded an average hydrodynamic size and PdI of 85.1 and 0.152 for TiO_2_NP, and 112.9 and 25.0 for CeO_2_NP. The hydrodynamic size of TiO_2_NP in complete cell culture media (10% FBS) was proven to be concentration-dependent, increasing from 49.8 at 0.75 mg/L to 108.6 at 75 mg/L. On the contrary, PdI decreased with increasing concentration, indicating that although the size of the aggregates is increasing, the homogeneity of the dispersion is improving. Upon incubation for 24 h (37 °C; 5% CO_2_), the size of the aggregates slightly increased with time. For CeO_2_NP, hydrodynamic size did not change with concentration. It should be noted that due to the low concentrations in the study (ng/L range), DLS may not be capable of detecting the particles; hence, these results must be interpreted with reservation. However, the slight red-shift in UV-Vis spectra (from 300 to 305 nm) seems to indicate the formation of aggregates of CeO_2_NP upon addition to cell culture media. Zeta potential was 41.1 ± 1.6 mV and 14.9 ± 0.7 mV for TiO_2_NP and CeO_2_NP dispersion in water, respectively. These results indicate that although both NP have a net surface positive charge, TiO_2_NP are more stable in solution that CeO_2_NP. It was impossible to measure zeta-potential of NPs in cell culture media, as the conductivity of the latter was too high to perform a standard measurement.

### 3.2. Cytotoxicity of Single Exposures

**Figure 7:**
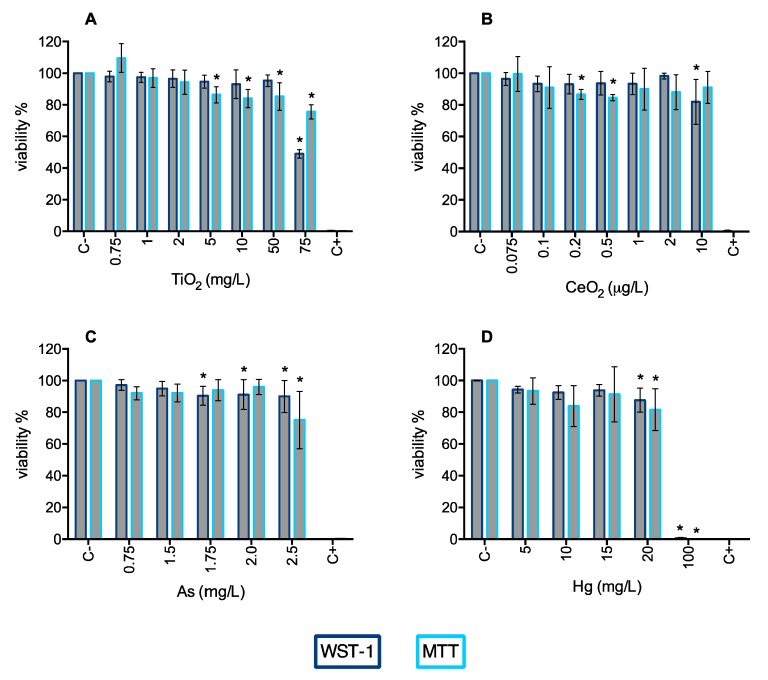
Viability of A549 cells 24 h post-exposure to TiO_2_NP (**A**), CeO_2_NP (**B**), As (**C**), and Hg (**D**) assessed by WST-1 (dark blue bars) and MTT (light blue bars) assays. C− negative control; C+: positive control (Triton X-100 1%). Values are expressed as mean ± standard deviation (n = 4, each experiment in triplicate). Statistical significance of samples compared to C− is indicated by * (One-way ANOVA).

To assess possible cytotoxic effects on A549, cells were treated with different concentrations of TiO_2_NP (0.75–75 mg/L; equivalent to 0.23–22.73 μg/cm^2^), CeO_2_NP (0.75–10 μg/L; equivalent to 0.23–3.03 ng/cm^2^), As (0.75–2.5 μg/L) and Hg (5–100 mg/L) for 24 h and cell viability was measured by MTT and WST-1 assays. Both assays were used to evaluate the mitochondrial function as an indirect measurement of cell viability (Figure 7).

Based on the results of WST-1, a significant decrease in cell viability was observed for TiO_2_NP 75 mg/L, where after 24 h exposure A549 viability was 48.9% (Figure 7A). Statistically, the decrease in cell viability caused by exposure to CeO_2_NPs 10 µg/L is considered to be significantly different (Figure 7B); however, it should be noted that it is a reduction of circa 20%, indicating only slight toxicity of CeO_2_NPs at this concentration. The same reservation has to be made for As and Hg induced cytotoxicity to A549 cells. Statistical analysis indicated that there is significant cytotoxicity caused by As in the concentration range of 1.7–2.5 mg/L (Figure 7C), and Hg at 20 and 100 mg/L. However, from a practical point of view, the magnitude of the effect is not that noticeable, with the exception of Hg 100 mg/L which completely inhibits cell viability (Figure 7D).

When assessed by MTT assay, a significant increase in cytotoxicity for cells exposed up to 5 mg/L of TiO_2_NP, and for both the 0.2 and 0.5 µg/L CeO_2_NP concentrations was observed (Figure 7A,B, respectively). Arsenic and Hg treatments decreased the mitochondrial activity only for the highest concentrations (Figure 7C,D).

The results of MTT showed larger standard deviation errors than those obtained with WST-1 assay, which can also account for the observed statistical differences, but that may not translate in an effective decrease of viability. This demonstrates the high heterogeneity and fluctuation of results, probably due to the nature of the assay. While MTT and WST-1 assays are analogous in the way they both assess cell viability indirectly through the generation of formazan by metabolically active cells, the latter is more stable and does not require the dissolution of the formazan crystals; therefore, is less laborious and less prone to human error. In this way, the higher coefficient of variance observed in the MTT assay could be explained by the need to remove the exposure solutions prior to the addition of DMSO. Fragile cells can incidentally be removed in this process, hence overestimating the decrease in viability and increasing the variance among replicates.

### 3.3. Cytotoxicity of Binary Mixtures

Based on single exposure results, two concentrations of each substance were chosen to further investigate the effects of binary mixtures in A549 cells: (1) a concentration was no toxic effects were seen; (2) A concentration where the substance-induced observable toxic effects. Hence, TiO_2_NP 1 and 75 mg/L; CeO_2_NP 0.1 and 10 µg/L; As 0.75 and 2.5 mg/L; and Hg 10 and 20 mg/L. Exceptionally, the third concentration of Hg was tested to assess if the NPs had any inhibitory effects in the mortality caused by 100 mg/L.

**Figure 8:**
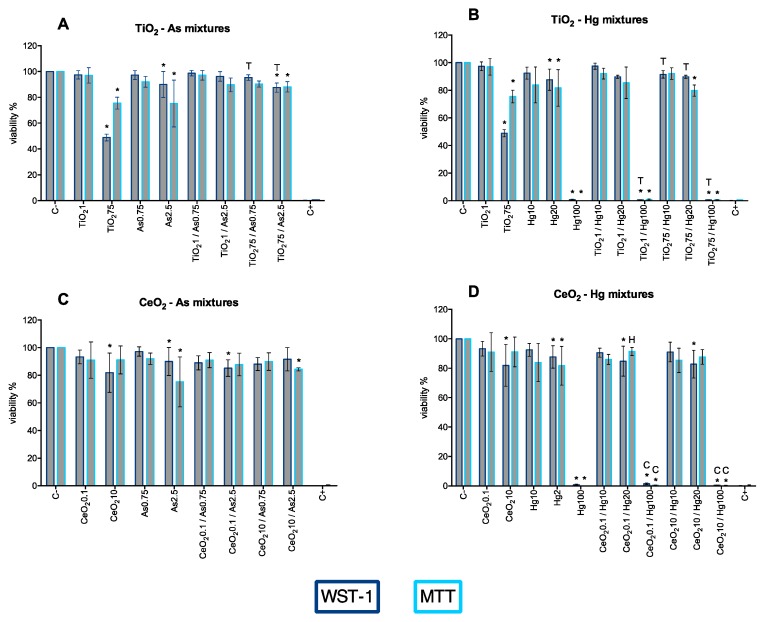
Viability of A549 cells 24 h post-exposure to binary mixtures of NP and metals-TiO_2_NP-As (**A**), TiO_2_NP-Hg (**B**), CeO_2_NP-As (**C**), CeO_2_NP-Hg (**D**), assessed by WST-1 (dark blue bars) and MTT (light blue bars) assays, compared to single exposure (left bars). All concentrations expressed in mg/L with the exception of CeO_2_NP which is expressed in µg/L. C−: negative control; C+: positive control (Triton X-100 1%). Values are expressed as mean ± standard deviation (n ≥ 3, each experiment in triplicate). Statistical significance of samples compared to C− is indicated by *; also indicated is statistical difference compared to NP (Titanium T; Cerium C) and/or metal (Arsenic A; Mercury H) (One-way ANOVA; *p* ≤ 0.05).

Overall, no significant differences were observed between mixtures and the corresponding single exposures. However, some exceptions were observed. The cell relative viability was significantly decreased for TiO_2_NP75/As2.5 co-exposure, compared with control, for both WST-1 and MTT assay (Figure 8A). Compared with a single exposure, cell toxicity decreased to a much lower level upon combined treatment of TiO_2_NP 75 mg/L with As (both concentrations—Figure 8A). Regardless of the concentration of TiO_2_NP used, Hg 100 mg/L still completely inhibited cell viability (Figure 8B). When compared with a single exposure to TiO_2_NP 75 mg/L, the viability of A549 increases when co-treated with Hg 10 or 20 mg/mL (Figure 8B), suggesting an antagonistic effect of Hg in TiO2NP toxicity at higher concentrations. No significant differences were observed for any of the CeO_2_NP/As mixtures when compared to a single exposure to these substances, but a significant decrease in viability was observed for CeO_2_NP0.1/As2.5 (WST-1) and CeO_2_NP10/As2.5 (MTT) when compared to the negative control. Despite the statistical analysis indicating there is a significant increase in toxicity for the co-exposure group CeO_2_NP/Hg20 (WST-1), regardless of the concentration of CeO_2_NP used, the observed effect is not biologically relevant. Similarly to what was observed for single exposure to Hg 10 mg/L, its mixture with CeO_2_NP (both concentrations) caused a complete inhibition of A549 viability, which is also responsible for the significant difference obtained when compared to CeO_2_NP (Figure 8D). The mixture CeO_2_NP0.1/Hg20 resulted in less toxicity than exposure to this concentration of Hg alone.

**Figure 9:**
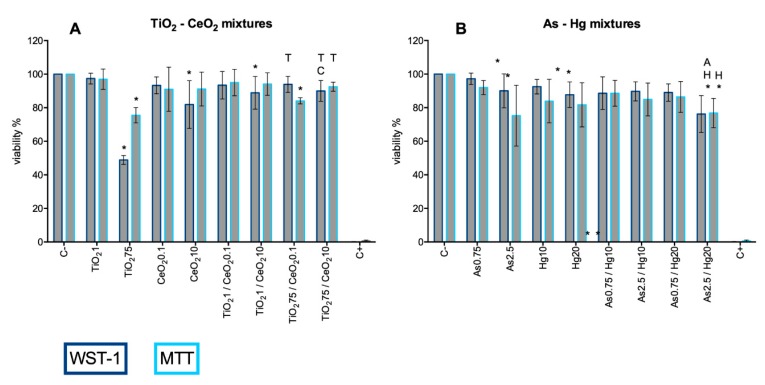
Viability of A549 cells 24 h post-exposure to binary mixtures of TiO_2_-CeO_2_ nanoparticles (**A**) and As-Hg (**B**) assessed by WST-1 (dark blue bars) and MTT (light blue bars) assays, compared to single exposure (left bars). All concentrations expressed in mg/L with the exception of CeO_2_NP which is expressed in µg/L. C−: negative control; C+: positive control (Triton X-100 1%). Values are expressed as mean ± standard deviation (n ≥ 3, each experiment in triplicate). Statistical significance of samples compared to C− is indicated by *; also indicated is statistical difference compared to Titanium T; Cerium C; Arsenic A; and Mercury H (One-way ANOVA; *p* ≤ 0.05).

Additionally, the cytotoxicity induced by TiO_2-_CeO_2_ nanoparticle mixtures and As-Hg mixtures was explored (Figure 9). The most notable finding for NPs mixture was the significant reduction of TiO_2_NP 75 mg/L cytotoxicity when in the presence of CeO_2_NP (Figure 9A). The analysis of Figure 9B indicates that, at the highest concentrations, As and Hg appear to have a cumulative cytotoxic effect with a significant decrease in cell viability when compared to negative control and single exposure to the compounds.

### 3.4. Long-Term Single Exposure

**Figure 10:**
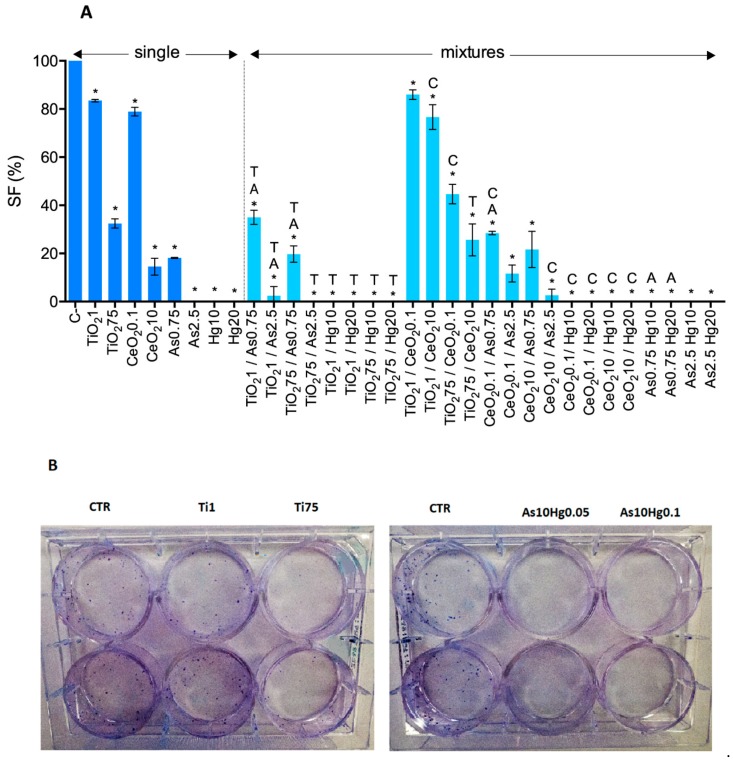
Clonogenic assay: (**A**) cytotoxicity of A549 cells exposed to TiO_2_NP, CeO_2_NP, As, and Hg alone or as binary mixtures for 7 days, determined by the clonogenic assay. Values are expressed as mean ± standard deviation (n = 3, each experiment in duplicate). All concentrations expressed in mg/L with the exception of CeO_2_NP which is expressed in µg/L. Statistical significance of samples compared to C− is indicated by *; also indicated is statistical difference compared to Titanium T; Cerium C; Arsenic A; and Mercury H (one-way ANOVA; *p* ≤ 0.05). (**B**) Representative images of Clonogenic assay after staining of the colonies.

To investigate the proliferative capacity of cells under exposure to single or binary mixtures for longer periods (7 days), the clonogenic assay was used. For NP single exposures, data showed a concentration-dependent decrease in the SF of A549 cells (Figure 10A). Exposure to TiO_2_NP 1 and 75 mg/L caused a decrease of 16% and 68% in colony formation, respectively, when compared to the negative control (visible in Figure 10B right). A similar pattern was observed for CeO_2_NP, where inhibition of colony formation was higher at the highest concentration (an 85% decrease for 10 µg/L) than at the lowest concentration (a 21% decrease for 0.1 µg/L). Cells exposed to As 0.75 mg/L exhibit an SF of 18%, while for 2.5 mg/L it was observed a complete inhibition of colony formation. The major long-term toxic effects were shown for Hg. Results indicate that upon exposure to both concentrations of Hg, there was no capacity of a single cell to grow into a colony.

### 3.5. Long-Term Exposure to Binary Mixtures

After 7-days treatment with TiO_2_NP + As, the SF of A549 was significantly lowered when compared to control (between 65–100% decrease). The major level of toxicity was observed at the highest doses of TiO_2_NP and As, where no colony formation was detected (Figure 10B). Compared to exposure to these compounds alone these results show that the long-term cytotoxicity of TiO_2_NP was magnified, while for As the opposite was observed, indicating that the co-exposure of these compounds influences each other’s toxicity. The mixture of CeO_2_NP and As showed the same pattern of behavior as described for TiO_2_NP and As, with a particular decrease in As toxicity even for the highest concentration. Overall, CeO_2_NP have the capacity to reduce As toxicity, while their own is increased. The analysis of results of NP binary mixture (TiO_2_NP + CeO_2_NP) indicates that TiO_2_NP has a higher influence in CeO_2_NP toxicity than the opposite, but no tendency in decrease or increase of toxicity can be observed. While no significant difference was observed for TiO_2_NP 1 mg/L mixtures, an increased toxicity of TiO_2_NP 75 mg/L was achieved when mixed with CeO_2_NP 10 µg/L (SF decreased from 32 to 26%), while the latter’s toxicity was decreased (15% to 26% SF); the same can be observed when mixed with TiO_2_NP 1 mg/L. In the combination TiO_2_NP 75 mg/L with CeO_2_NP 0.1 µg/L, titanium’s toxicity is decreased (from 32% to 45% SF) and cerium is increased (from 79% to 45% SF). Arsenic mixtures with nanoparticles result in increased toxicity for both TiO_2_NP and CeO_2_NP, while arsenic’s toxicity is significantly reduced in almost every mixture. As shown in Figure 10B, no combination of Hg with other substances had the ability to reduce its toxicity, with Hg-containing mixtures inducing the strongest reduction of clonogenic survival.

### 3.6. Effect of Singles Exposures on Cell Cycle

**Figure 11:**
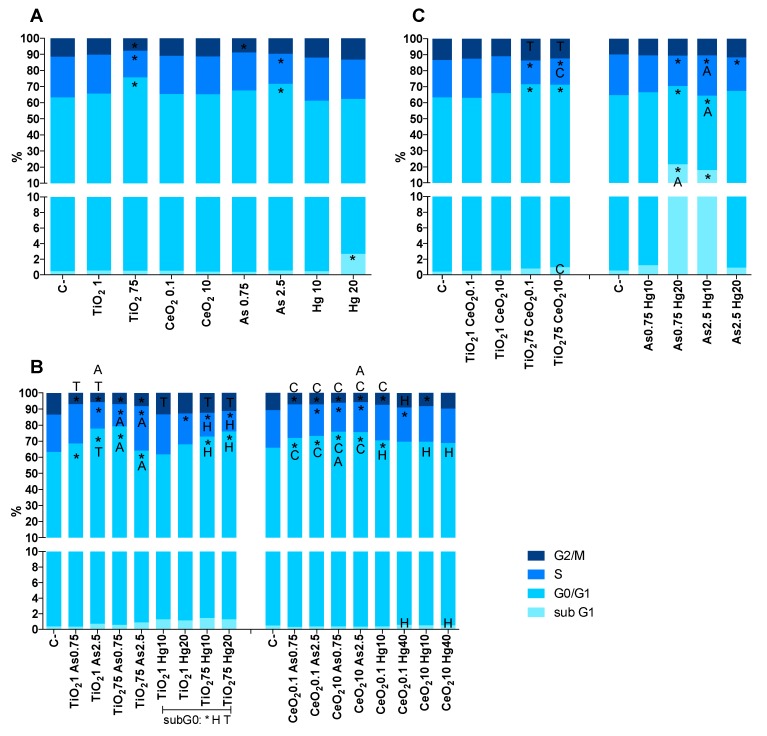
Cell cycle and sub*G*1 A549 cell population observed after 24 h exposure to (**A**) single substances; (**B**) binary mixtures of NP-metals; and (**C**) binary mixtures of TiO_2_NP and CeO_2_NP and As-Hg. Values are expressed as mean ± standard deviation (n = 3, each experiment in triplicate). All concentrations expressed in mg/L with the exception of CeO_2_NP which is expressed in µg/L. Statistical differences (One-way ANOVA; *p* ≤ 0.05) against *: negative control; T: TiO_2_NP single; C: CeO_2_NP single; A: arsenic single; and H: mercury single exposure.

Cell cycle changes after single exposures were investigated by Flow Cytometry (FCM) (Figure 11). Control cells show a typical profile of proliferation at the time point with most dominant subpopulations at *G*0/*G*1 (62%) and at *G*2/*M* (11%) together with a high rate of cells at *S*-phase. The results of cell cycle analysis do not show a significant alteration on cell cycle dynamics for any NP concentrations up to the highest concentrations of TiO_2_NP (75 mg/L). The highest concentration of TiO_2_NP induced a significant increase in *G*0/*G*1 (76%) cell population at the expense of the two other phases, particularly *S*-phase (16%). In this study, As induced cell cycle alterations in a dose dependent manner. Where the lower concentration of As (0.75 mg/L) decreased the *G*2/*M*-phase, the higher concentration increased *G*0/*G1* subpopulation (72%), at the expense of *S*-phase (Figure 11A). On the contrary of what was expected, Hg induced fewer alterations on cell cycle dynamics of A549 cells, the only observed effect was a marked elevation of cells at sub-*G*1 phase (often associated with cells with DNA break/damage) for Hg 20 mg/L.

### 3.7. Effect of Binary Exposures on Cell Cycle

Figure 11 shows the distribution of the cell cycle in the different samples. Similarly to the results of NP singles exposures for the highest concentration of TiO_2_NP, the co-exposure TiO_2_NP75/CeO_2_NP0.1 and TiO_2_NP75/CeO_2_NP10 affected the cell cycle progression causing accumulation of A549 in *G*0/*G*1 phase and decrease of the S population when compared to the negative control. However, when compared with the single exposure to TiO_2_NP 75 mg/L, the *G*2/*M* was not affected, suggesting an antagonistic effect of CeO_2_NP in TiO_2_NP toxicity. The analysis of Figure 11B indicates that, compared to negative control, the cell cycle dynamics were affected by the co-exposure to As/Hg, except for As0.75/Hg10. For both As0.75/Hg20 and As2.5/Hg10 there was a noticeable increase in sub-*G*1 cell population (up to 8%), which was the same observed alteration in the cell cycle caused by the exposure of Hg alone, and a decreased *G*0/*G*1-phase. Although the group exposed to As0.75/Hg20 showed a decreased *G*2/*M*-phase, the mixture As2.5/Hg10 induced an arrest on the same phase of the cell cycle. The only observed effect at the highest concentrations of As/Hg was a decrease in the percentage of cells in the *S*-phase.

When compared to the negative control, the cell cycle data of cells co-exposed to CeO_2_NP/As, showed a significant increase in the resting phase *G*0/*G*1 along with a decrease in the percentage of cells in S phase, regardless of the concentration. Compared to exposure to these compounds alone, the cell cycle dynamics were significantly altered for CeO_2_NP exposure, suggesting an antagonistic effect of As in CeO_2_NP toxicity when present in the mixture.

**Figure 12:**
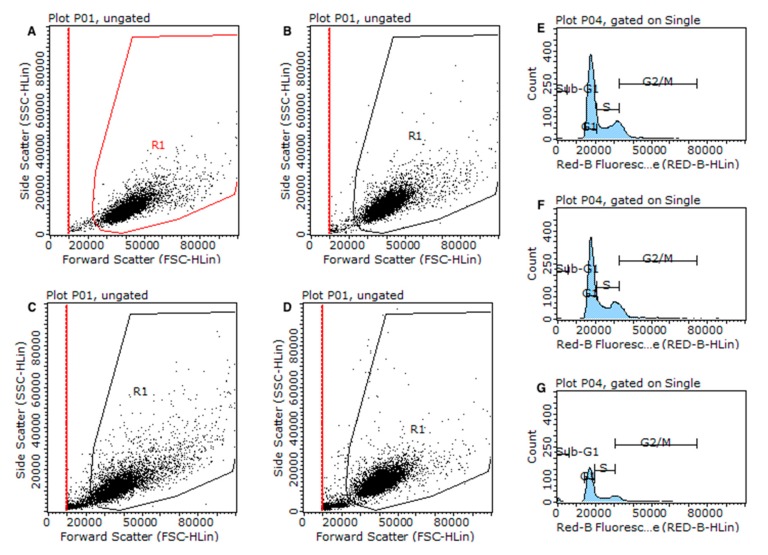
Representative images of viable cell gating (side scatter (SSC) vs. forward scatter (FSC) (**A**–**D**) and histograms of cell cycle analyzed by flow cytometry (Sub-*G*1, *G*0/*G*1, S, and *G*2/*M*) in A549 cells after exposure to CeO_2_NP 10µg/L (**E**), Hg 20 mg/L (**F**) and the mixture CeO_2_NP10/Hg20 (**G**). The *y*-axis shows the number of cells counted and the *x*-axis shows an increasing amount of PI incorporation/cell (right). Experiments performed in triplicate yielded similar results.

Overall, all concentrations of the mixture CeO_2_NP/Hg induced a cell cycle arrest at the resting phase *G*0/*G*1. Additionally, CeO_2_NP0.1/Hg10 and CeO_2_NP10/Hg10 decrease the percentage of cells in *G*2 phase. The concentration of CeO_2_NP used in this study seems to have some influence on the alterations induced by Hg. Figure 12 shows the representative gating strategies to select live cells, as well as the cell cycle histogram. In this image, it is clear that the cell population gated after exposure to the mixture CeO_2_NP10/Hg20 reflects the results of both the Hg20 and CeO_2_NP10 singles exposure.

TiO_2_NP seems to reduce Hg toxicity, decreasing the number of cells in phase Sub-*G*1 (Figure 11C), but significant accumulation of cells in *G*0/*G*1 (decrease of the *S*-phase) was also observed, especially for the highest concentration of TiO_2_NP (75 mg/L). The cell cycle data confirmed that A549 exposure to TiO_2_NP/As resulted in cell cycle perturbation for all concentrations, leading to a predominant number of cells accumulated in *G*0/*G*1 phase and a decrease in the percentage of cells in phase *G*2/*M*. Compared to the effects of As and TiO_2_NP single exposure (Figure 11A), co-exposure of these compounds is reflected in an increase of cells in sub-*G*1 phase. Additionally, the group TiO_2_NP1/As2.5 and TiO_2_NP75/As0.75 decreased the number of cells in *S*-phase, while in turn the group TiO_2_NP75/As2.5 induced an arrest in the same phase. Previously, in a single exposure of As and TiO_2_NP data (Figure 11A), almost no toxicity has been observed for the lowest concentrations, therefore the effect of the mixture TiO_2_NP/As seems to be additive, inducing higher alterations in the cell cycle, which could ultimately lead to cell death.

## 4. Discussion

The alveolar epithelium constitutes a large surface area of lungs providing surface for gas exchange, is key in regulating this organ’s homeostasis, and is a major target of environmental insults because of its proximity to inhaled toxicants. Therefore, the maintenance of alveolar surface integrity is vital [45,46,47]. Hence, the present study focused on the assessment of the potential cytotoxicity caused by exposure of A549 human cell line to legacy (mercury and arsenic) and emergent (TiO_2_NP and CeO_2_NP) environmental contaminants. The high removal capacity of As and Hg by these metal-based nanomaterials has been previously demonstrated [39,48,49,50,51]. While this can have a positive environmental clean-up effect, it can be inferred that the NP-metal complexes will interact with organisms as well.

Prior to experiments, TiO_2_ and CeO_2_ nanoparticles were characterized for their physicochemical properties, and demonstrated to be crystalline, pure materials. Once the nanomaterials were dispersed in complete cell culture media (10% FBS) the observed enlarged size (compared to dispersion in water) indicates the formation of agglomerates/aggregates and/or interaction of NPs with serum proteins with the formation of the protein corona. This phenomenon has been well-described in the literature and strongly influences cellular uptake and toxicity, since after its formation, it is the protein outer layer of the corona that interacts with the cell. In turn, the protein corona characteristics are dependent on NPs’ physicochemical characteristics and concentration, as well as culture media and serum composition [52,53,54].

Cell viability was first assessed after exposure of A549 cells to the individual substances. The range of concentrations chosen intends to be realistic, based on current estimations of these contaminants in the environment [37,38].

The state-of-the-art shows that combined exposure to multiple chemicals often results in different toxicity to that of the individual substances [55]. Hence, it is imperative to recognize and address the risks from exposure to multiple chemicals in a more systematic way, as consistently drawn attention by the Joint Research Centre (JRC) [56], and Scientific Committee on Health and Environmental Risks (SCHER), Scientific Committee on Consumer Safety (SCCS), Scientific Committee on Emerging and Newly Identified Health Risks (SCENIHR) [57]. With the foreseeing increased exposure of humans to legacy (e.g., metals) and emerging (e.g., nanomaterials) contaminants, a thorough understanding of the potential toxicity resulting from acute and long-term exposure to unintentional mixtures of these contaminants is required. This affirmation is supported by the results presented in this study showing that, even at low concentrations, mixtures of As, Hg, TiO_2_NP and CeO_2_NP may result in different toxicity to A549 cell line than the individual counterparts, particularly after long-term (seven-day) exposure.

At the tested concentrations, after 24 h exposure, cell viability is only affected at the highest concentrations but there is a tendency for a concentration-dependent decrease observed in all substances. Nevertheless, since it is a reality that humans are facing an ever-increasing contaminated environment, these results seem to suggest a greater risk of developing an adverse outcome. This is more evident when analyzing the longer exposure period where the prolonged exposure resulted in significant inhibition of cell proliferation even at low concentration of the contaminants, more evident for arsenic and mercury.

The results obtained after exposure to TiO_2_NP alone are in accordance with previously published data, where inhibition of cell viability and cell cycle alterations are only observed at concentrations higher than the predicted environmental concentrations [21,58]. Effects of combined 24 h exposure of this nanomaterial with either As or Hg proved to be concentration-dependent. At the lowest concentration (1 mg/L), mixtures of TiO_2_NP/As and TiO_2_NP/Hg induced a slow-down of the cell cycle progression and an increase in the resting phase (*G*0/*G*1), indicative of DNA synthesis inhibition as well. This cytostatic effect does not imply a cytotoxic effect as observed in data from Figure 8 (viability assessment). On the other hand, when TiO_2_NP concentration was increased to 75 mg/L, the significant viability inhibition observed for TiO_2_NP alone was diminished. Moreover, TiO_2_NP partially mitigated the adverse effects of As (but not Hg) after a 7-days exposure period (no adverse outcome was observed for As exposure alone after 24 h exposure), indicating that the magnitude of joint toxicity is not only concentration-, but also, time-dependent. One possible explanation for the observed decreased toxicity is that the hypothetical adsorption of As at the NP surface results in lower effective dose and bioavailability of both substances in the exposure medium. A second explanation could be that NPs compete with the metalloid for the binding sites on the cell membrane, promote their intra- and extracellular degradation and/or modulate enzymatic activities that regulate toxicity, resulting in limited contaminant cytotoxicity [59,60]. To confirm this hypothesis, further studies are being conducted. To be noted that not even the highest concentration of TiO_2_NP was able to mitigate Hg high cytotoxicity at seven-day exposure.

A similar pattern to that described for TiO_2_NP-metal(loid) mixtures was observed for CeO_2_NP in co-exposure with As and Hg. It is noteworthy to mention that, according to the results presented in Figure 10A, the sub-*G*1 phase caused by exposure to a concentration of 20 µg/L Hg alone is significantly decreased by co-exposure with CeO_2_NP, indicating a possible long-term protective effect of this material in A549 cells. CeO_2_NP, at low concentration, have been described to exert a cytoprotective effect by other authors [37,38].

Limited data of multiple NPs exposure is available [55]. The results of this study show that CeO_2_NP yielded an antagonistic cytotoxicity on TiO_2_NP, but more evident effects were observed for the longer exposure period, where the mixture toxicity was altered. In this case, the joint toxicity seems to reflect the average toxicity of each NP alone. Since zeta-potential indicates that both NPs have the same charge, these particles will not have a tendency to co-aggregate or co-precipitate and, in turn, will compete for adsorption sites in the cell wall [7].

A cumulative effect could be observed when A549 cells were exposed to a mixture of the highest concentrations of As and Hg (Figure 9B). Although both substances alone disturbed the cell cycle dynamics (Figure 11A), the analysis of Figure 11C seems to indicate that, at the highest concentrations, the mixture of As and Hg does not cause cell cycle changes, while a significant increase in the population in sub-*G*1 was verified at lower concentrations. This increase in sub-*G*1 population, also observed for exposure to Hg alone (more evident at 20 mg/L; Figure 11A), and without significant alterations in other phases, may be an indicator of severe and irreparable damage, and consequently to a lower number of viable cells after exposure, as indicated by WST-1 assay (viability decrease after 24 h) and the clonogenic assay where the surviving factor was always null if Hg was present in the exposure medium. This fragility of the cells may have consequences in the protocol. As previously discussed, cells exposed to the highest concentration of Hg seem to become fragile and likely to be lost during the preparation steps for cytometric analysis, resulting in an underestimation of the cell cycle alterations. Indeed, a detailed analysis of the data revealed that after 24 h of exposure, the number of cells in the various treatments with As and Hg was different. The number of cells acquired by the cytometer was 200 cells/µL for control, 103 cells/µL for As0.75Hg20, 110 cells/µL for As2.5Hg10 and 58 cells/µL for As2.5Hg20. This means that the analysis of data and interpretation of results should be done carefully and conscientiously of the steps involved in the protocols.

## 5. Conclusions

Due to natural and anthropogenic sources, there is a complex mixture of contaminants in the environment, to which humans are exposed to and impacted by. Currently, available data on the joint toxicity of environmental contaminants is very limited. This work explored the effects of single and combined exposures to TiO_2_NP, CeO_2_NP, As, Hg in A549 cells, a contribution to the knowledge of mixture toxicology. In this study, results show that, after 24 h exposure, cell viability was affected at the highest concentrations, while prolonged exposure caused inhibition of cell proliferation even at low concentration of all substances, more evidently for arsenic and mercury. Arsenic induced mitochondrial toxicity, decreased cell proliferation, and caused cell cycle disruption. Upon co-exposure with CeO_2_NP or TiO_2_NP toxicity was reduced. The major toxic effects were shown for Hg exposures. Results indicate that regardless of the concentration of TiO_2_NP and CeO_2_NP used, Hg reduced the mitochondrial activity and completely inhibit cell proliferation after long-term exposure. Additionally, is noteworthy to mention that co-exposure with CeO_2_NP decreased the number of cells in sub-*G*1 phase caused by exposure to Hg alone, indicating a possible long-term protective effect of this material in A549 cells. A cumulative effect could be observed when A549 cells were exposed to a mixture As/Hg, but analyzing the cell data, the mechanism underlying its toxicity seems to be distinct. Therefore, to explore the potential mechanisms of the combination of legacy and emergent environmental contaminants further studies will be conducted to focus on uptake, signaling and cell death pathways in vitro and in vivo, as well as to explore genotoxic and oxidative outcomes, supported by characterization of the NPs-metal(loid) mixtures. The results here presented clearly show that mixtures of nanoparticles and metals may have different toxic behavior than their individual counterparts and that it is imperative to better understand the risk associated with co-exposure to environmental contaminants.

## Supplementary Materials

The following are available online at https://www.mdpi.com/2079-4991/10/3/447/s1, Figure S1: EDX spectra of TiO_2_NP from 0 to 20 keV (A) and from 1 to 20 keV (B), Figure S2. SAED pattern of TiO_2_NP, Figure S3. EDX spectra of CeO_2_NP, Figure S4. SAED pattern of CeO_2_NP.
