# Supplementary material for: Unravelling the Potential Cytotoxic Effects of Metal Oxide Nanoparticles and Metal(Loid) Mixtures on A549 Human Cell Line
Source: Nanomaterials (Basel). 2020 Mar 2;10(3):447. doi: 10.3390/nano10030447 (PMC7153484; doi:10.3390/nano10030447)
Supplement: Supplementary file 1 [file nanomaterials-10-00447-s001.pdf]

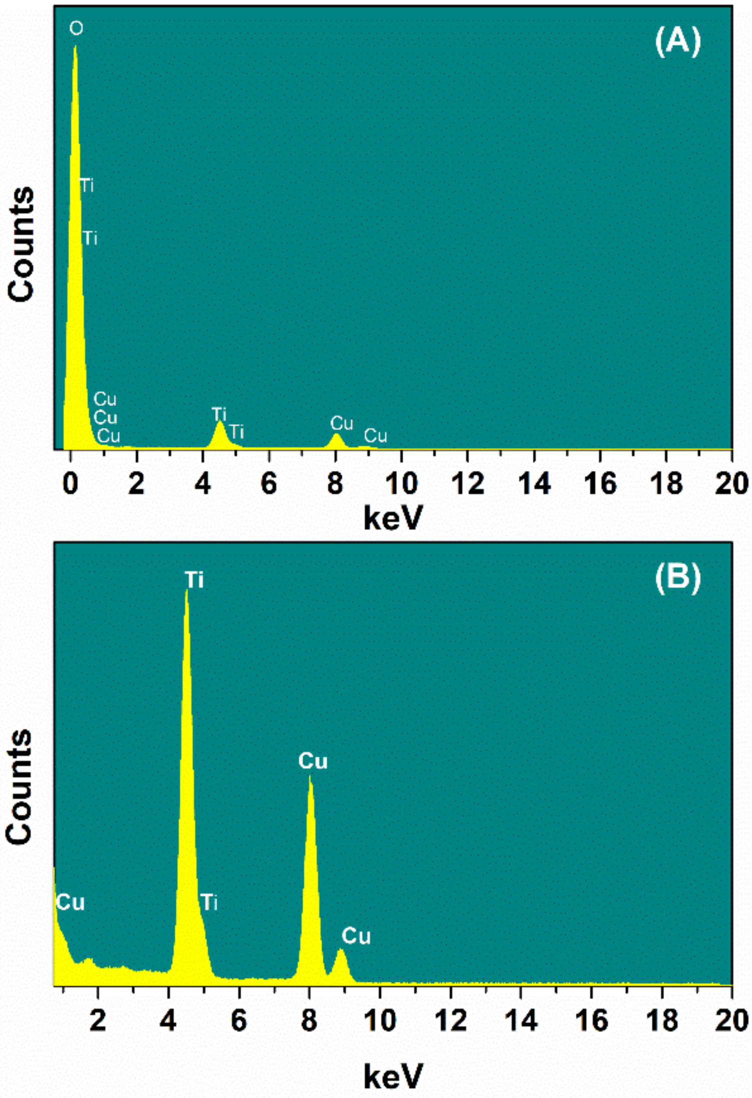

Figure S1. EDX spectra of TiO_2NP from 0 to 20 keV (A) and from 1 to 20 keV (B).

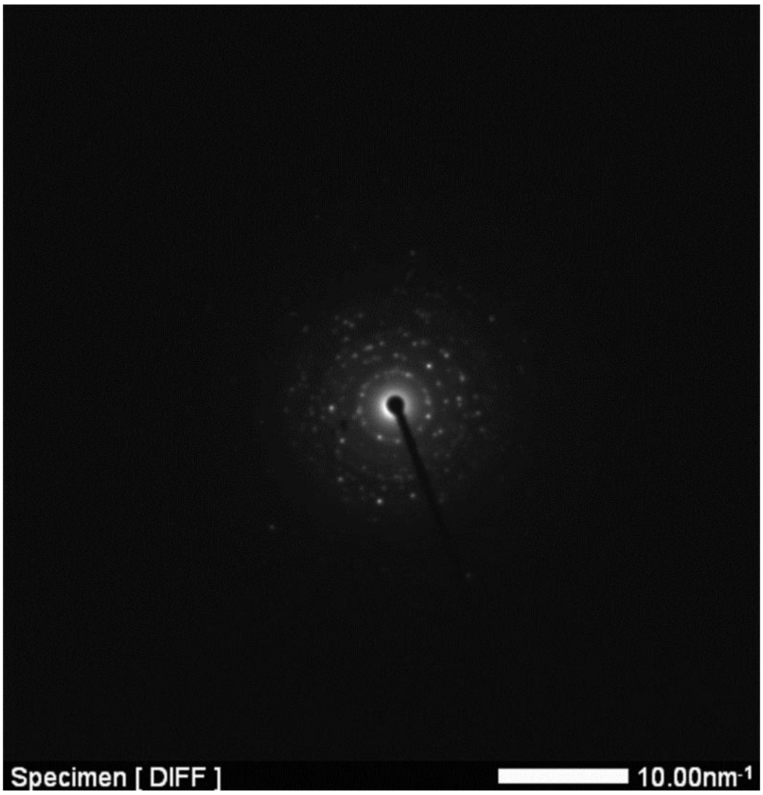

Figure S2. SAED pattern of TiO_2NP.

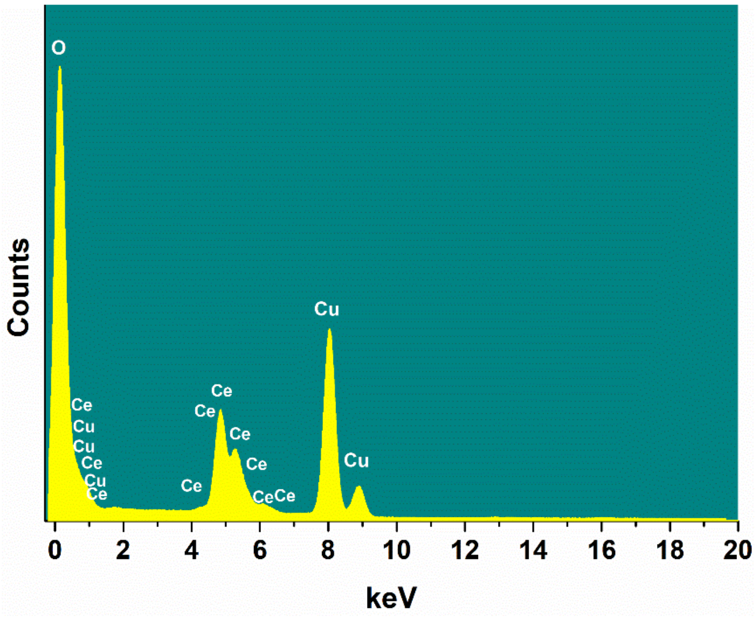

Figure S3. EDX spectra of CeO_2NP.

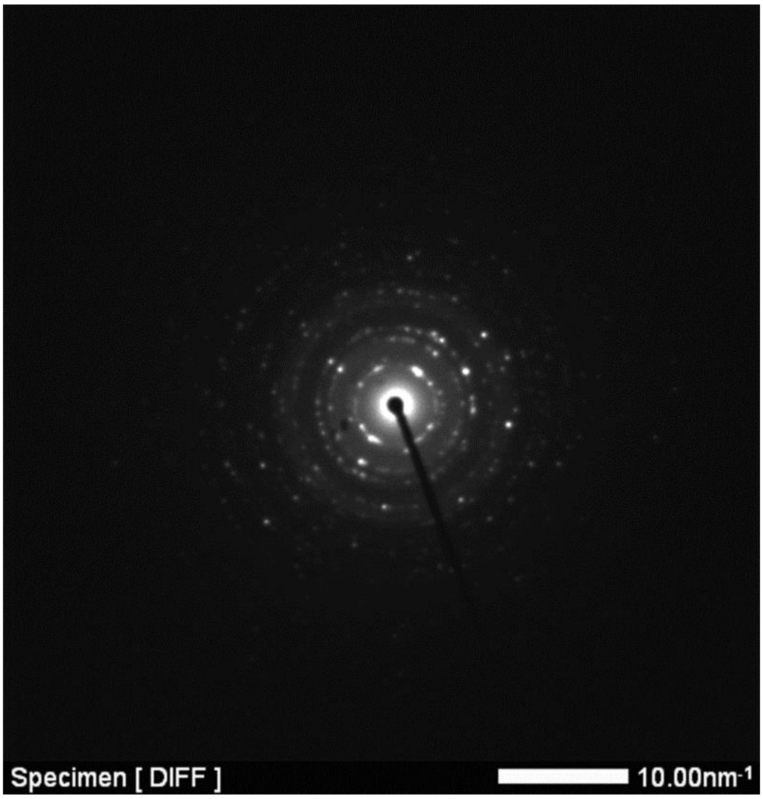

Figure S4. SAED pattern of CeO_2NP.
